# Comparison of the effect of self-selected and prescribed moderate-intensity aerobic exercise on state anxiety symptoms and affective responses in young women: a randomised crossover clinical trial design

**DOI:** 10.1017/neu.2025.10022

**Published:** 2025-07-14

**Authors:** Naiane Silva Morais, Vinnycius Nunes de Oliveira, Rizia Rocha-Silva, Wellington Fernando da Silva, Ricardo Borges Viana, Carlos Alexandre Vieira, Mario Hebling Campos, Marilia Santos Andrade, Rodrigo Luiz Vancini, Katja Weiss, Thomas Rosemann, Beat Knechtle, Claudio Andre Barbosa de Lira

**Affiliations:** 1 Faculdade de Educação Física e Dança, Universidade Federal de Goiás, Goiânia, Brazil; 2 Instituto de Educação Física e Esportes, Universidade Federal do Ceará, Fortaleza, Brazil; 3 Departamento de Fisiologia, Universidade Federal de São Paulo, São Paulo, Brazil.; 4 Centro de Educação Física e Desportos, Universidade Federal do Espírito Santo, Vitória, Brazil; 5 Medbase St, Gallen Am Vadianplatz, St. Gallen, Switzerland; 6 Institute of Primary Care, University of Zurich, Switzerland

**Keywords:** Mental disorders, Psychological stress, Aerobic exercise, Emotional regulation, Complementary therapies

## Abstract

**Objective:**

To investigate the effect of physical exercise intensity on state anxiety symptoms and affective responses.

**Methods:**

Twenty-one healthy women (mean age: 23.6 ± 5.4 years) participated in three sessions: self-selected intensity exercise, moderate-intensity prescribed exercise, and a nonexercise control session. Before each session, participants were exposed to unpleasant stimuli. State anxiety symptoms and affective responses were assessed pre- and post-stimulus exposure and pre- and post-sessions. A two-way repeated measures ANOVA tested state anxiety, while the Friedman test analysed affective responses.

**Results:**

Time significantly affected state anxiety symptoms [*F* (2,0) = 25.977; *P* < 0.001; *η* ^2^ *_p_* = 0.565]. Anxiety increased post-stimulus (*P* < 0.001) and decreased after all sessions. No significant differences were found between exercise and control conditions. Time also significantly influenced affective responses [*χ* ^2^ (8.0) = 62.953; *P* < 0.001; Kendall’s *W*: 0.375]. Affective responses decreased post-stimulus (*P* = 0.029) and significantly increased after both exercise sessions (*P* < 0.001) but remained unchanged in the control session (*P* = 0.183).

**Conclusions:**

Although state anxiety increased after unpleasant stimuli in all conditions, reductions following exercise sessions were comparable to the nonexercise session. However, both exercise sessions uniquely improved affective responses, highlighting their potential for emotional recovery after unpleasant stimuli.

## Significant outcomes

- The unpleasant stimuli strategy to experimentally elevate state anxiety symptoms was effective.
- The state anxiety symptoms decreased similarly after all sessions, both physical exercise and nonexercise control.
- The affective responses increased only after both physical exercise sessions.

## Limitations

- The sample was limited to young, healthy women, restricting generalizability to other groups.
- Only acute effects of a single exercise session were evaluated, not long-term impacts.
- Relevant biomarkers (e.g., cortisol, serotonin, endorphin) were not analysed.

## Introduction

Anxiety is an adaptive emotion from an evolutionary standpoint, as it enables individuals to manage dangerous situations (Crocq, 2015). However, when exacerbated, it can lead to anxiety disorders, which affect millions of people worldwide, greatly impacting their quality of life (Higgins *et al*., 2021). According to the World Health Organization (WHO, 2017), in 2015, approximately 264 million people (3.6% of the global population) suffered from anxiety disorders. As a result of the COVID-19 pandemic, this number increased to 374 million by 2020 (Santomauro *et al*., 2021). In Brazil, the country with the highest prevalence of anxiety disorders, 9.3% of the population suffers from these disorders (WHO, 2017).

Women are more affected than men by anxiety disorders across all stages of life (WHO, 2017). Women are more likely than men to experience anxiety-related conditions, owing to a complex interplay of biological, psychological, and social factors (NIMH, 2016; WHO, 2017). Therefore, studies focused on coping strategies for anxiety disorders among women are warranted.

The treatment for anxiety disorders involves pharmacological and nonpharmacological approaches. The pharmacological approach uses drugs, such as selective serotonin reuptake inhibitors, serotonin-norepinephrine reuptake inhibitors, benzodiazepines, antidepressants, and anxiolytics (Bandelow *et al*., 2017). However, nonpharmacological treatments, such as psychotherapy (Bandelow *et al*., 2017), acupuncture (Yang *et al*., 2021), and physical exercise (Connor *et al*., 2023), are effective in the management of anxiety symptoms.

Acute and chronic physical exercises exert their anxiolytic effects through physiological and psychological mechanisms, such as the release of endorphins, improved regulation of neurotransmitters (e.g., serotonin and dopamine), and increasing brain-derived neurotrophic factor (Anderson and Shivakumar, 2013). Additionally, physical exercise promotes exposure to some physiological symptoms, such as elevated heart rate, which decrease anxiety sensitivity and increased self-efficacy (van de Vegte *et al*., 2019). Physical exercises can confer a distraction from stressors from daily activities (Anderson and Shivakumar, 2013).

Although the general benefits of physical exercise on mental health are well-documented (Mahindru *et al.*, 2023), there is a gap in understanding the impact of exercise intensity, particularly self-selected versus prescribed intensities, on anxiety symptoms. When individuals are free to choose their physical exercise intensity, they often report greater enjoyment, higher sense of control, and higher motivation to engage in future exercise sessions (Parfitt *et al*., 2006; Oliveira *et al*., 2015). These findings support the self-determination theory, which posits that autonomy is a fundamental psychological need to enhance intrinsic motivation and overall well-being (Berger and Motl, 2000).

Despite the important effects of self-selected intensity exercise, prescribed intensity physical exercise has traditionally been used to investigate the benefits of physical activity on mental health (Connor *et al*., 2023; Mahindru *et al.*, 2023). Although this approach is effective in ensuring that individuals meet the necessary thresholds for physiological adaptations, such as improved cardiovascular fitness, it may not always align with individual preferences. Therefore, it could be perceived as less enjoyable or more strenuous, which can negatively impact mood and reduce adherence to exercise programmes (Ekkekakis, 2009). Hence, there is a need for studies investigating this issue.

The high prevalence of anxiety disorders in women and the potential of physical exercise as a non-pharmacological tool to manage anxious symptoms necessitate further research to identify how self-selected intensity exercise influences anxiety symptoms. This is particularly relevant for young women, as they are disproportionately affected by anxiety disorders and may benefit from personalised and autonomy-supportive physical exercise interventions.

To cover this knowledge gap, this study was conducted to examine effect of type of physical exercise (self-selected intensity aerobic physical exercise, prescribed aerobic physical exercise at moderate intensity, and a control session without physical exercise) on anxiety symptoms and affective responses in young women after a single session exposure unpleasant stimuli. To increase state anxiety in healthy subjects, we followed a standardised protocol that involved exposing them to unpleasant images from the International Affective Picture System (IAPS) (see Methods for details). Secondarily, this study examined how type of exercise (self-selected intensity aerobic physical exercise vs prescribed intensity aerobic physical exercise) influenced enjoyment and intention for future involvement after a single exercise session following exposure to unpleasant stimuli. The central research hypothesis was that self-selected intensity exercise would promote a greater reduction in state anxiety symptoms and a greater increase in affective responses than the prescribed intensity exercise and the control session.

## Materials and methods

### Participants

A total of 26 participants were initially recruited, through direct invitation, e-mail or social media, based on the following inclusion criteria: i) being female and ii) aged between 18 and 40 years. The following exclusion criteria were adopted: i) being in the menstrual period at the time of recruitment; ii) a history of irregular menstrual cycle; iii) having any contraindication for physical exercise (assessed by Physical Activity Readiness Questionnaire - PAR-Q); iv) having any type of cardiac, endocrine, orthopaedic, or metabolic dysfunction (self-reported assessment); v) use of pharmacological anxiolytics; vi) being illiterate, as participants needed to answer inventory and questionnaire; and vii) failure to complete all stages of the study. After random selection, five participants were excluded due to their unavailability to complete all sessions. Thus, 21 young women were included in the study (Table 1). Regarding their ethnicity, four participants self-identified as black, seven as mixed-race, and eight as Caucasian, but two did not provide a response.

During anamnesis, the researcher asked participants whether they used any contraceptive method and, if so, what type they used. Fourteen reported not using any, while seven did (four used oral contraceptives and three had an intrauterine device).

**Table 1.** Characteristics of the participants (*n* = 21)

	Mean ± SD	Min–Max	CI (95%)	*P*-value^a^
**Variables**				
Age (years)	21.00 [7.00]^b^	18.00–34.00	21.13–26.01	0.003
Body mass (kg)	61.53 ± 9.45	49.60–83.90	57.23–65.84	0.200
Body height (m)	1.62 ± 0.61	1.47–1.72	1.59–1.64	0.717
BMI (kg/m^2^)	23.38 ± 2.93	18.30–30.10	22.05–24.72	0.546
Maximal speed from GET (km/h)	11.85 ± 1.93	8.00–15.00	10.97–12.73	0.295
Trait-anxiety symptoms	46 ± 14	23–72	40–53	0.910

BMI: body mass index. GET: grade exercise testing. SD: standard deviation. Min: minimum. Max: maximum. CI: confidence interval.

a : Shapiro–Wilk test.

b : median [interquartile range].

### Study design

A within-group randomised crossover clinical trial design was conducted to examine the effects of type of physical exercise (self-selected vs prescribed intensity of aerobic physical exercise) on state anxiety symptoms in women. Participants completed four laboratory visits, interspersed by a minimum 24 h. All visits took place under the same laboratory settings and at the same time of day for each participant, minimising variability due to environmental factors and daily physiological and psychological fluctuations (Difrancesco *et al*., 2019).

During the first visit, the participants underwent anamnesis, assessment of trait anxiety symptoms, anthropometric evaluation, and graded exercise testing. In subsequent visits, participants were randomly subjected to three experimental conditions: a self-selected intensity session, a prescribed exercise session at moderate intensity according to the maximal heart rate percentual (%HRmax) criterion of the American College Sports Medicine guideline (Garber *et al*., 2011), and a nonexercise control session. Each session (the control session and the main phase of the physical exercise session) lasted 30 min. In accordance with standard exercise testing and presciption guidelines, each physical session included a 5-minute warm-up and a 5-minute cool-down to ensure participant safety and adherence to best practices (McGowan *et al*., 2015 Van Hooren and Peake, 2018). Thus, the total duration of the physical exercise sessions was 40 minutes (5 minutes of warm-up, 30 minutes of exercise, and 5 minutes of cool-down).

Before each experimental condition, participants were exposed to unpleasant images from the IAPS (Costa *et al*., 2022) for approximately 30 min. This strategy is effective for examining the impact of an intervention on anxiety symptoms in nonclinical populations (Smith, 2013; Viana *et al*., 2021; Costa *et al*., 2022). Additionally, it helps create a more homogeneous sample, as anxiety disorders are multifaceted and often cause significant variability in symptom intensity among patients.

All images were rated according to a paper-and-pencil version of the Self-Assessment Manikin (SAM) (Lang *et al*., 2008), an instrument that rates the participants’ pleasure, arousal, and dominance after viewing each image. State anxiety symptoms and affective responses were assessed at three time points in all sessions: pre-IAPS, post-IAPS, and postsession. Enjoyment and intention for future involvement were evaluated after the physical exercise sessions (self-selected and prescribed intensities). The heart rate was continuously monitored throughout each session.

Participants were instructed to attend the sessions wearing appropriate clothing, to refrain from engaging in vigorous physical activity, and to avoid consuming alcohol, stimulants, tobacco, or illicit drugs on the day before the sessions. They were also advised to maintain their usual dietary habits throughout the study period.

Exercise sessions were randomised using the online tool Research Randomiser (Urbaniak and Plous, 2013), with counterbalanced sequences to ensure that participants did not consistently start with the same intervention, thereby minimising potential order effects.

### Experimental procedures

### Anamnesis and anthropometric assessment

Anamnesis was performed using the PAR-Q (Canadian Society for Exercise Physiology, 2002) to screen for potential health risks related to physical activity. Body mass was measured using a digital balance (Omrom, HN-289, USA) and height was measured using a wall stadiometer (Caumaq, Brazil). The body mass index was calculated by dividing the body mass (kg) by the square of body height (m^2^).

### Graded exercise testing

All participants underwent a graded exercise test to determine their maximum heart rate (HRmax). This value was used as a reference to prescribe moderate exercise intensity in the prescribed intensity session (Garber *et al*., 2011). The test was performed on a motorised treadmill (ATL, Inbramed, Brazil) with a 0% slope. The protocol consisted of three stages adapted from de Lira *et al*., (2013): i) a 5-min warm-up at 6 km/h; ii) incremental stages, with the speed increasing by 1 km/h each minute until the participant reached exhaustion; and iii) a 5-min cool-down at 4 km/h. The heart rate was monitored during the test using a heart rate monitor (Vantage M, Polar, Finland) synchronised with a heart rate sensor (H10, Polar, Finland).

### Physical exercise session at a self-selected intensity

The self-selected intensity session consisted of walking/running on a motorised treadmill (ATL, Inbramed, Brazil) with a 0% slope. The entire protocol lasted 40 min, comprising a 5-min warm-up at 6 km/h, a 30-min walking/running at the participant’s self-selected intensity, and a 5-min cool down at 4 km/h. Before the self-selected physical exercise session, the researcher instructed the participants with the following statement “Think of an intensity that you would like to exercise on your daily routine.” During the session, participants were free to adjust the treadmill speed every 5 min, with the option to increase or decrease the speed by 1 km/h or maintain their current pace (Parfitt *et al*., 2006). The heart rate was continuously monitored during the session using a heart rate monitor (Vantage M, Polar, Finland) synchronised with a heart rate sensor (H10, Polar, Finland).

### Prescribed physical exercise session at a moderate intensity

The prescribed physical exercise at moderate intensity session consisted of walking/running on a motorised treadmill (ATL, Inbramed, Brazil) with a 0% slope. The entire protocol lasted 40 min, comprising a 5-min warm-up at 6 km/h, a 30-min walking/running at moderate intensity, and a 5-min cool down at 4 km/h. The level of moderate intensity was determined using the HRmax obtained from the graded exercise testing. It was calculated as 64–76% of HRmax (Garber *et al*., 2011) and the heart rate was monitored and regulated by the researcher to ensure it remained within that target zone. The heart rate was continuously monitored during the session using a heart rate monitor (Vantage M, Polar, Finland) synchronised with a heart rate sensor (H10, Polar, Finland).

### Nonexercise control session

The nonexercise control session took 30 min during which a participant was seated on a chair without reclining. Participants were instructed not to use their smartphones, fall asleep, or speak to the researcher. The heart rate was continuously monitored during the session using a heart rate monitor (Vantage M, Polar, Finland) synchronised with a heart rate sensor (H10, Polar, Finland).

### Unpleasant stimuli

A set of 69 unpleasant images (Costa *et al*., 2022) from IAPS (Lang *et al*., 2008) was used to induce a state of pathological anxiety in the participants. The IAPS is a widely used and internationally standardised tool that includes a broad array of visual stimuli. It comprises hundreds of high-resolution colour images designed to evoke emotional responses and encompasses nearly all facets of everyday situations (e.g., sports, fashion, landscapes, and violence) (Lang *et al*., 2008). The IAPS has been used in multiple studies to mimic an anxiety state (Smith, 2013; Viana *et al*., 2021; Costa *et al*., 2022).

All images were rated on a paper-and-pencil version of SAM, which includes three domains, pleasure, arousal, and dominance, each assessed on a 9-point rating scale represented by manikins. Each of these dimensions is represented by five graphical figures in the form of a human manikin, with rectangles between them indicating intermediate markers. The pleasure dimension ranges from “happy” to “sad,” the arousal dimension from “very tense” to “calm,” and the dominance dimension from “out of control” to “in full control’ (Bradley and Lang, 2007; Crabbe *et al*., 2007; Lang *et al*., 2008). These domains reflect how participants felt after viewing each image.

### Outcome measurements

### State-trait anxiety symptoms

Anxiety symptoms were assessed using the Brazilian Portuguese version (Gorenstein and Andrade, 1996) of the State-trait Anxiety Inventory (STAI) (Spielberger *et al*., 1970). This instrument consists of two scales, namely, the state (acute symptoms) and trait (chronic symptoms). Each scale includes a 20-item self-report inventory, where each statement is rated on a 4-point Likert scale (1 to 4). Scores range from 20 to 80, with the following classifications: ≤30 indicate low anxiety, 31 to 49 indicate intermediate anxiety, and ≥50 indicate high anxiety (Spielberger *et al*., 1970). The internal consistency of the STAI is strong, with Cronbach’s alpha values of 0.93 for the state scale and 0.87 for the trait scale (Knight *et al*., 1983). The trait anxiety inventory was administered by a trained researcher during the participants’ first visit and was used to characterise them. The state anxiety inventory was also administered by a trained researcher during subsequent visits at three time points: pre-IAPS, post-IAPS, and postsession.

### Affective responses

Affective responses were assessed using the Brazilian Portuguese version (Alves *et al*., 2019) of the Feeling Scale (Hardy and Rejeski, 1989). Using this instrument, which consists of an 11-point bipolar scale, ranging from 5 (very bad) to + 5 (very good), participants rated how they felt “at the present moment.” In pilot testing, the Feeling Scale exhibited correlations ranging from 0.51 to 0.81 on the Valence scale of the Self-Assessment Manikin and from 0.41 to 0.59 on the Valence scale of the Affect Grid (Landuyt *et al*., 2000). The scale was administered during both the physical exercise and control sessions at three time points: pre-IAPS, post-IAPS, and postsession.

### Enjoyment

Enjoyment was assessed using the Brazilian Portuguese version (Alves *et al*., 2019) of the Physical Activity Enjoyment Scale (PACES) (Kendzierski and DeCarlo, 1991). PACES is an 18-item self-reported instrument, with a 7-point Likert scale, ranging from 1 to 7. Participants rated how they felt “during the physical exercise they performed” (Kendzierski and DeCarlo, 1991). Scores ranged from 18 to 126 points, with higher scores indicating greater enjoyment. PACES was administered after both physical exercise sessions.

### Intention for future involvement

The intention for future involvement was assessed using a single scale ranging from 0 to 100%, with 10% increments (Fox *et al*., 2000). The scale has anchored phrases at 0% (not interest at all), 50% (moderately interest), and 100% (very interested) (Fox *et al*., 2000); higher scores indicate greater intention for future involvement. This test was administered after both physical exercise sessions.

### Statistical analysis

The Shapiro–Wilk test was used to assess data for normality. The data were visually inspected for outliers using boxplots and identified outliers were treated using the winzorization method, which replaces outlier values with the nearest maximum or minimum values identified after their exclusion (Kwak and Kim, 2017; Goss-Sampson, 2022). Three outliers were identified: ID11 (post-exercise in the prescribed session) and ID2 and ID14 (post-control session), and were appropriately managed. The base-10 logarithm (log10) of the state anxiety symptoms followed a normal distribution; therefore, we performed two-way repeated measures analysis of variance (ANOVA) with two within-group factors (session × time) to test for significant differences in the log10-transformed values of the state anxiety symptoms among the sessions (self-selected, prescribed physical exercise, and nonexercise control). The Bonferroni post hoc analysis was used for pairwise comparisons of means. The effect size was reported as partial eta squared (η^2^_p_), classified as follows: <0.01 (trivial), 0.01 (small), 0.06 (medium), and 0.14 (large) (Goss-Sampson, 2022).

As affective responses did not follow a normal distribution, the Friedman test was used to test for significant differences among the three sessions. The Conover post hoc was used for pairwise comparisons.

As enjoyment and intention for future involvement data did not follow a normal distribution, a Wilcoxon signed ranked test was used to test for significant differences between the two physical exercise sessions. The effect size used for this test was the rank biserial correlation (*r*_B_), which was classified as follows: <0.10 (trivial), 0.10 to 0.29 (small), 0.30 to 0.49 (medium) and≥0.5 (large) (Munro, 1986).

As the heart rate exhibited a normal distribution, the student paired *t*-test was used to test for significant differences between the physical exercise sessions. The effect size used for this test was the Cohen’s d, which was classified as follows: <0.20 (trivial), 0.20–0.49 (small), 0.50–0.79 (medium), and≥0.8 (large) (Munro, 1986).

A significance level of 5% was adopted. Data that followed a normal distribution were expressed as mean ± SD, mean differences, and 95% confidence intervals (CI), whereas data that did not follow a normal distribution were presented as median and interquartile range (IQR), median differences, and 95%CI. All statistical analyses were performed using Jeffrey’s Amazing Statistic Program (version 0.18.1.0, University of Amsterdan, Netherlands).

## Results

### State anxiety symptoms

The two-way repeated measures ANOVA indicated that time had a significant effect on state anxiety symptoms [F (2,40) = 25.977; *P* < 0.001; η^2^_p_ = 0.565; “large”]. However, there was no significant effect of session [F (2,40) = 1.833; *P* = 0.173; η^2^_p_ = 0.084; “medium”] or interaction between session and time [F (4,80) = 1.370; *P* = 0.252; η^2^_p_ = 0.064; “medium”] on state anxiety symptoms. Table 2 presents the descriptive statistics for state anxiety by session and timepoint, including log-transformed values (used for statistical analysis) and raw data values to facilitate interpretation of the actual scores.

**Table 2.** Descriptive statistics by session and timepoint of state anxiety assessment (n = 21)

State-anxiety		Self-selected session (Mean ± SD)	Prescribed session (Mean ± SD)	Control session (Mean ± SD)
Pre-IAPS	Log-transformed values^a^	1.553 ± 0.130	1.569 ± 0.094	1.587 ± 0.124
	Raw data values	37.238 ± 11.022	37.905 ± 8.012	40.143 ± 11.065
Post-IAPS	Log-transformed values^a^	1.647 ± 0.123	1.662 ± 0.126	1.645 ± 0.110
	Raw data values	46.048 ± 12.639	47.714 ± 13.398	45.524± 11.250
Post-session	Log-transformed values^a^	1.516 ± 0.101	1.559 ± 0.091	1.562 ± 0.095
	Raw data values	33.714 ± 8.119	36.714 ± 8.498	38.190 ± 11.048

a Data expressed in log10 of the scores from the state anxiety assessment. SD: standard deviation.

**Figure 1.**
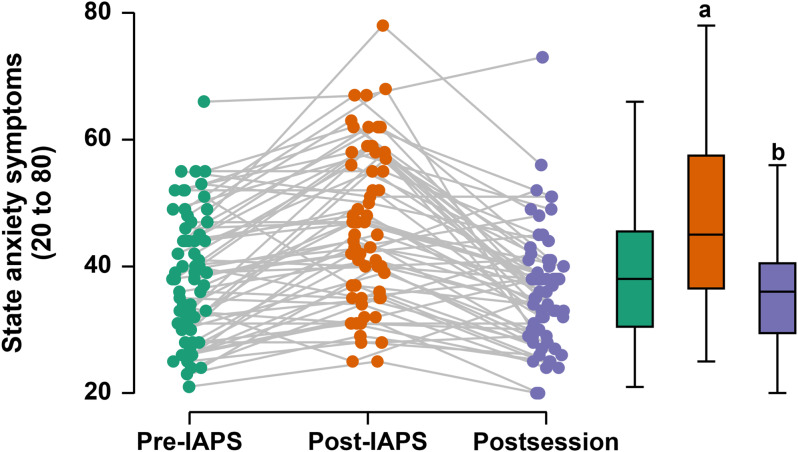
Effect of time on state anxiety symptoms in the participants (n = 21). **a:** higher than pre-IAPS time (all sessions); **b:** lower than post-IAPS time (all sessions). **IAPS**: international affective picture system.

After the Bonferroni post hoc correction, considering the time-points of state anxiety symptoms assessment, there was a significant increase in state anxiety symptoms after the unpleasant stimuli (post-IAPS) [mean difference = 0.082 (95% CI: 0.043 to 0.120); *P* < 0.001; Cohen’s *d* = 0.732; “medium”]. In contrast, state anxiety symptoms significantly decreased after each session [mean difference = −0.105 (95% CI: −0.144 to − 0.067); *P* < 0.001; Cohen’s *d* = 0.945; “large”]. Fig. 1 shows the effect of time on the state anxiety symptoms in the participants.

A post hoc power analysis was performed using the software G*Power (version 3.1.9.6; University of Kiel, Germany). With a moderate correlation for repeated measures (r = 0.5) and a partial eta squared (η^2^p) of 0.064 (converted to an effect size F of 0.2614882) for the interaction between the three conditions and time, a sample size of 21 participants provided a statistical power of >95%, indicating that the sample size was adequate.

### Affective responses

The Friedman test indicated that time had a significant effect on participants’ affective responses (χ^2^ (8.0) = 62.953; *P* < 0.001; Kendall’s W: 0.375). Table 3 presents the descriptive statistics for affective responses by session and timepoint.

**Table 3.** Descriptive statistics by session and timepoint of affective responses assessment (n = 21)

Affective responses	Self-selected session (Mean ± SD)	Prescribed session (Mean ± SD)	Control session (Mean ± SD)
Pre-IAPS	3.286 ± 1.419	2.905 ±1.513	2.810 ±1.887
Post-IAPS	1.190 ± 2.089	0.857 ± 2.197	1.095 ± 2.234
Post-session	3.667 ±1.426	3.238 ± 1.480	2.333 ± 1.623

SD: standard deviation.

**Figure 2.**
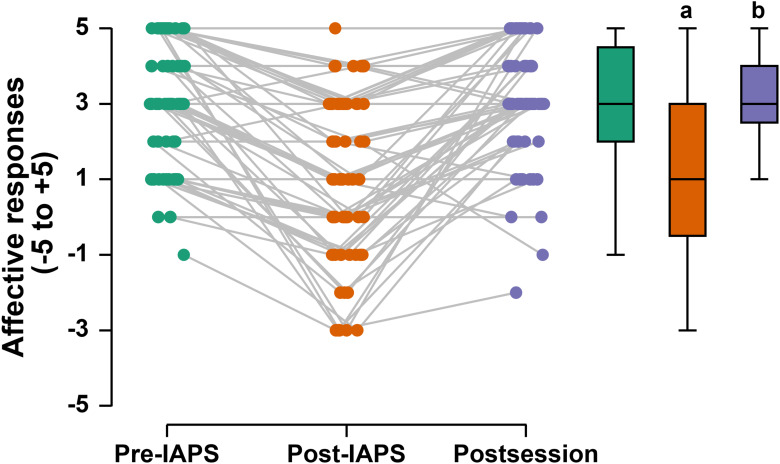
Effect of time on affective responses in the participants (n = 21). **a:** lower than pre-IAPS time (all sessions); **b:** higher than post-IAPS time (only physical exercise sessions). **IAPS**: international affective picture system.

The Conover post hoc correction showed that, in the self-selected session, affective responses at the post-IAPS time point (1[3] a.u.) were significantly lower than those at the pre-IAPS time point [3(3) a.u.), *T* (160) = 4.2; *P* = 0.001]. This was followed by a significant increase after the self-selected session (4[2] a.u.), *T* (160) = 5.6; *P* < 0.001] relative to the post-IAPS time point. In the prescribed session, affective responses at the post-IAPS time point (1[3] a.u.) were significantly lower than those at the pre-IAPS time point [3(2) a.u.), *T* (160) = 4.3; *P* < 0.001]. This was followed by a significant increase after the prescribed session (3[1] a.u.), *T* (160) = 5.3; *P* < 0.001], relative to the post-IAPS time point. In the nonexercise control session, affective responses at the post-IAPS time point (1[3] a.u) were significantly lower than those at the pre-IAPS time point [3[1] a.u.), *T* (160) = 4.5; *P* < 0.001]. However, there was no significant change after the control session [3 [2] a.u., *T* (160) = 2.8; *P* = 0.183], relative to the post-IAPS time point. Fig. 2 shows the effect of time on the participants’ affective responses. Fig. 2 shows the effect of time on the participant’ affective responses.

### Enjoyment

The Wilcoxon test showed no significant difference in enjoyment between the self-selected and prescribed physical exercise sessions (W = 108.500, *P* = 0.133, r_B_ = 0.418; “medium”). The median enjoyment scores for the self-selected and prescribed physical exercise sessions were 96 [IQR: 27] and 93 [IQR: 25], respectively, corresponding to 76.2% and 73.8% of the maximal score, respectively.

### Intention for future involvement

The Wilcoxon test showed no significant difference in the intention for future involvement between the self-selected and prescribed physical exercise sessions (W = 48.000, *P* = 0.189, r_B_ = 0.455; “medium”). The median intention for future involvement scores for the self-selected and prescribed physical exercise sessions were 80 [IQR: 30] and 80 [IQR: 40], respectively.

### Heart rate

The Student’s paired samples *t*-test showed that the mean HR for the self-selected physical exercise session (144 ± 15 bpm, 74.6% of HRmax) was significantly higher [t (20) = 3.841, *P* = 0.001, Cohen’s *d* = 0.838; “large”] than that for the prescribed physical exercise session (133 ± 9 bpm, 68.7% of HRmax).

## Discussion

The primary objective of this study was to examine the effect of nature of physical exercise (self-selected intensity aerobic physical exercise, prescribed aerobic physical exercise at moderate intensity, and a control session without physical exercise) on state anxiety symptoms and affective responses in young women after a single session. The secondary objective of this study was to compare enjoyment and intention for future involvement after a single session between self-selected intensity aerobic physical exercise and prescribed intensity aerobic physical exercise at moderate intensity. The main findings of this study were that time had a significant effect on state anxiety symptoms, but “session” or the interaction between “session and time.” had no effect. Additionally, there was a significant effect of “time” on affective responses.

The state anxiety symptoms of participants increased after exposure to unpleasant stimuli in all physical exercise sessions, indicating that the strategy of using unpleasant images from the IAPS to elevate state anxiety symptoms was effective. These findings are in line with those of previous studies. Viana *et al*. (2021) recorded extreme evidence of changes in healthy women’s state anxiety after they viewed IAPS unpleasant images. Costa *et al*. (2022) report that state anxiety and state anger were higher in postunpleasant session in women than before exposure. For men, only state anxiety was higher in the postunpleasant session. These findings reveal that exposure to unpleasant pictures can act as an anxiogenic stimulus to induce experimental anxiety.

Contrary to our hypothesis, the state anxiety symptoms decreased similarly after all sessions. These results complement those of previous studies. Knapen *et al*., (2009) found that both the self-selected and prescribed intensity (50% of maximal heart rate reserve) were effective in reducing state anxiety symptoms. Viana *et al*. (2017) recorded a significant reduction in state anxiety among participants after physical exercise. Morais *et al*. (2022) demonstrated that a dance exergame session reduced state anxiety symptoms, whereas traditional aerobic exercise did not. A common limitation in these studies was the lack of a nonexercise control group to account for random error, potentially introducing bias into the results. Thus, our findings are more robust, and show no difference between the acute exercise session and the control session.

The levels of affective responses in participants decreased after exposure to unpleasant stimuli across all sessions, but increased only after both physical exercise sessions, with no significant difference between the sessions. Again, these findings suggest that the anxiogenic effect due to exposure to unpleasant stimuli from the IAPS image is transitory. Santos *et al*. (2023) found no significant difference in affective responses between participants who had undergone high intensity functional training session and those who had undergone high-intensity continuous training session, and both were classified as “good.” Oliveira *et al*. (2015) report that prescribed exercise sessions elicited affective responses similar to those observed in the self-selected sessions. Based on a meta-analysis, Oliveira, *et al*. (2015) concluded that the difference in affective responses between the self-selected and prescribed sessions depends on the intensity of the prescribed session.

In this study, although the mean HR differed significantly between the self-selected and prescribed exercise sessions, the intensity classification, according to the American College of Sports Medicine guidelines, remained within the same moderate level (64–76% of HRmax) (Garber *et al*., 2011). The fact that both exercise sessions had the same duration and that the same intensity (moderate), might explain the absence of a significant difference between the physical exercise sessions.

Regarding enjoyment and intention for future involvement, there was no significant difference between self-selected and prescribed physical exercise sessions. Studies exploring enjoyment across various physical exercise modalities have reported different results. Morais *et al*. (2022) found that enjoyment during the exergame session was greater than that during the treadmill aerobic physical exercise. Soylu *et al*. (2021) reported that the high-intensity interval training group had higher enjoyment scores than the moderate-intensity continuous training group. Focht *et al*. (2015) found that participants had greater intention for future involvement with the self-selected loads than with prescribed loads. There are several possible factors to explain the results regarding enjoyment and intention for future involvement in this study. The continuous modality, similar intensity, and identical duration of both self-selected and prescribed physical exercise sessions in this study may have contributed to these outcomes.

This study had several notable strengths. First, it used a within-subject experimental design with clearly defined conditions—self-selected intensity, prescribed intensity exercise sessions, and a nonexercise control session—allowing for a balanced comparison between exercise sessions and enabling a relevant analysis of the acute effects of exercise on state anxiety symptoms. Second, the inclusion of a nonexercise control condition helped to control the effects of possible random extraneous factors. Although the study makes some relevant contributions, several limitations should be considered. First, the sample comprised young, apparently healthy women, which limits the generalizability of the findings to other population groups, such as individuals diagnosed with anxiety disorders. Further research should explore the effects of physical exercise in individuals with anxiety disorders. Second, the study focused on the acute effects of a single exercise session on state anxiety symptoms. Although the results suggest reduction in state anxiety symptoms after a single session, it is unclear whether these effects would be sustained or even amplified with regular exercise programmes. Therefore, chronic studies are necessary to determine whether physical exercise can provide long-term benefits in managing state anxiety symptoms. Third, this study did not include biochemical markers, such as cortisol, serotonin, and endorphin, which are relevant for a deeper understanding of the physiological mechanisms underlying these results. Further studies incorporating biomarker assessments would be valuable to enhance insights into the physiological processes associated with various exercise intensities. Fourth, the difference in total session duration among the experimental conditions. While the core aerobic exercise phase (self-selected or prescribed intensity) lasted 30 minutes, equivalent to the control condition, the physical activity sessions included an additional 5-minute warm-up and a 5-minute cool-down period, extending the total session time to 40 minutes. Although these segments involved low-intensity walking and are unlikely to have substantially influenced the primary psychological outcomes, participants remained exposed to the laboratory environment for an additional 10 minutes compared to the control group. Therefore, we cannot completely rule out the possibility that the additional time may have contributed to the observed effects, such as the return of affective measures to baseline levels.

## Conclusions

This study examined the impact of nature of physical exercise session (self-selected aerobic physical exercise, prescribed aerobic exercise at moderate intensity, and a nonexercise session) on the state anxiety symptoms among young women. The findings revealed that time had a significant effect on state anxiety symptoms, with no significant differences observed between the exercise sessions or between the exercise sessions and the control session. This suggests that whereas participants′ state anxiety symptoms increased after the unpleasant stimulus across all conditions, the extent of the subsequent reductions after both exercise sessions were not different from those after the nonexercise sessions. Additionally, both self-selected and prescribed exercise sessions exhibited a similar capacity to enhance affective responses after exposure to unpleasant stimuli. There were no notable differences in enjoyment or intention for future participation between the exercise types.

Although this study provides valuable insights, its limitations must be acknowledged. The study focused on a specific demographic group (young women) and the acute nature of the exercise interventions. Further research should aim to explore the effects of regular physical exercise in broader populations, including individuals diagnosed with anxiety disorders, to establish the long-term benefits of exercise in managing anxiety symptoms.

## Statement of Interest

No competing financial interests exist.
